# A Population-Based Acute Meningitis and Encephalitis Syndromes Surveillance in Guangxi, China, May 2007- June 2012

**DOI:** 10.1371/journal.pone.0144366

**Published:** 2015-12-03

**Authors:** Yihong Xie, Yi Tan, Virasakdi Chongsuvivatwong, Xinghua Wu, Fuyin Bi, Stephen C. Hadler, Chuleeporn Jiraphongsa, Vorasith Sornsrivichai, Mei Lin, Yi Quan

**Affiliations:** 1 Acute Infectious Disease Prevention and Control, Guangxi Zhuang Autonomous Region Center for Disease Prevention and Control, Nanning, Guangxi, China; 2 Epidemiology Unit, Faculty of Medicine, Prince of Songkla University, HatYai, Songkla, Thailand; 3 Division of Bacterial Disease, Centers for Disease Control and Prevention, Atlanta, Georgia, United States of America; 4 International Field Epidemiology Training Program (IFETP), Bureau of Epidemiology, Ministry of Public Health, Bangkok, Thailand; University of Texas Medical Branch, UNITED STATES

## Abstract

**Objectives:**

Acute meningitis and encephalitis (AME) are common diseases with the main pathogens being viruses and bacteria. As specific treatments are different, it is important to develop clinical prediction rules to distinguish aseptic from bacterial or fungal infection. In this study we evaluated the incidence rates, seasonal variety and the main etiologic agents of AME, and identified factors that could be used to predict the etiologic agents.

**Methods:**

A population-based AME syndrome surveillance system was set up in Guigang City, Guangxi, involving 12 hospitals serving the study communities. All patients meeting the case definition were investigated. Blood and/or cerebrospinal fluid were tested for bacterial pathogens using culture or RT-PCR and serological tests for viruses using enzyme-linked immunosorbent assays. Laboratory testing variables were grouped using factor analysis. Multinomial logistic regression was used to predict the etiology of AME.

**Results:**

From May 2007 to June 2012, the annual incidence rate of AME syndrome, and disease specifically caused by Japanese encephalitis (JE), other viruses, bacteria and fungi were 12.55, 0.58, 4.57, 0.45 and 0.14 per 100,000 population, respectively. The top three identified viral etiologic agents were enterovirus, mumps virus, and JE virus, and for bacteria/fungi were *Streptococcus sp*., *Cryptococcus neoformans* and *Staphylococcus sp*. The incidence of JE and other viruses affected younger populations and peaked from April to August. Alteration of consciousness and leukocytosis were more likely to be caused by JE, bacteria and fungi whereas CSF inflammation was associated with bacterial/fungal infection.

**Conclusions:**

With limited predictive validity of symptoms and signs and routine laboratory tests, specific tests for JE virus, mumps virus and enteroviruses are required to evaluate the immunization impact and plan for further intervention. CSF bacterial culture cannot be omitted in guiding clinical decisions regarding patient treatment.

## Introduction

Acute meningitis and encephalitis (AME) is a syndrome of central nervous system (CNS) infections, which could lead to fatality and neurological damages. The etiologic agents include viruses, bacteria, fungi and parasites, etc. Worldwide, around 200,000 viral encephalitis cases are reported annually [1]. At least 275,000 deaths from bacterial meningitis have been estimated to have occurred in all ages in 2010 [2]; around 166,100 of these being children aged under 5 years [3]. The permanent disability rate ranges between 25% to 50% after bacterial infections and 18% after viral infection [4]. With the concomitant use of immunosuppressive therapies and common HIV infection, the incidence of CNS mycosis has increased in the last two decades. Thus, there is a need to understand the infectious etiologies of AME.

More than 100 viruses and multiple bacteria species have been identified as AME etiology [5,6]. In China, Japanese encephalitis (JE) and *Neisseria Meningitidis (Nm*) are the most common notifiable causes of AME. In the 1970s, the annual incidence rate of JE and *Nm* were 21 and 60 per 100,000 population, respectively. After JE and *Nm* polysaccharide vaccines were introduced, the incidence rate for JE was reduced to less than 1 per 100,000 since 1996 and for *Nm* was reduced to 0.25 per 100,000 in 2010 [7,8]. Vaccines preventing both diseases as well as mumps and rubella have been included in the expanded programme on immunization (EPI) in China since 2008. However, apart from JE and *Nm*, there is no surveillance system for other causes of AME in China.

In 2006, an acute meningitis and encephalitis syndrome (AMES) surveillance project was launched in China, Bangladesh and India [9]. While the main protocol in China aimed to identify four vaccine preventable diseases (JE, *Nm*, *Haemophilus influenza type B* [*Hib*], *Streptococcus pneumoniae* [*Sp*]) and the overall incidence of AME, we expanded the attempt to identify the pathogens in more detail in order to facilitate prevention and control of these specific diseases in the future.

The specific objectives of this report include: (1) to determine the incidence rate, seasonality and the causative pathogens of AME, (2) to analyze the pattern of clinical manifestations of these infections and their relationship with the pathogens.

## Methods

### Study area

Guangxi is one of the four provinces in China that are involved in the AMES project. Guigang City, the sentinel site, is located in the southeast of Guangxi and had a total population of 4.1 million in 2007 [10]. There are five counties and 12 hospitals serving the whole area/population. Before the AMES project commenced, two JE outbreaks were reported in 1999 and 2003, the latter including 50 cases with 4 fatalities [11].

The AMES surveillance system was run from May 2007 until June 2012. Of the 12 hospitals available in the area, five with best facilities were set as sentinel hospitals, to which the other seven non-sentinel hospitals referred all AME patients.

### Case investigation and definition

The surveillance focused on admitted cases in pediatrics, neurology, internal medicine and infectious disease wards of the 12 hospitals. A patient was defined as an AME suspected case if he/she had symptoms and signs of CNS infection, including acute onset, plus at least one symptom of fever, headache or vomiting plus either meningeal signs or changes in mental status. Suspected cases detected at any of the seven non-sentinel site hospitals were referred for investigation at one of the five sentinel site hospitals where blood and cerebrospinal fluid (CSF) samples are taken. A suspected case was upgraded to a probable AME case after rabies, tetanus and cerebral malaria were ruled out. Both the blood and CSF samples underwent further tests to confirm the diagnosis.

A JE case was diagnosed if the blood or CSF IgM test result was positive and the subject had not been immunized against JE virus vaccine within the past 3 months. Likewise, other viral AME diagnoses were also based on IgM tests of blood specimens.

### Epidemiological investigation and laboratory test procedures

AME suspected cases were to be notified to the local Center for Disease Control and Prevention (CDC) within 24 hours. Epidemiological investigation was further carried out by local CDC staff using a structured questionnaire. Variables collected included vaccine history of JE, *Nm* and *Hib*, demographic and clinical information, and biochemistry test results.

For identification of etiologic agents, a subsample of CSF and blood specimens were cultured for bacteria at the sentinel hospital. Another subsample was tested for JE IgM using enzyme-linked immunosorbent assay (ELISA) at Guigang CDC. The remaining parts of the specimen were sent to Guangxi CDC for bacterial PCR (*Nm*, *Hib*, *Sp*) on the CSF specimen with standard methods [12] and other viral IgM tests on the blood specimen.

For cases having symptoms onset during 2007 and 2008, a battery of tests for other viral infections was sequentially carried out using viral specific IgM in preferential descending order: JE virus (Beixi Kit, Shanghai B&C Biological Technology Co., Ltd., China), enteroviruses (Coxsackievirus & ECHO virus [EV]; Institute Virion \Serion GmbH), herpes simplex virus (HSV; IBL International GmbH [IBL]), cytomegalovirus (CMV; IBL), rubella virus (RV; IBL), measles virus (MEV; IBL), mumps virus (MV; IBL), varicella-zoster virus (VZV; IBL), EB virus (EBV; IBL). In order to minimize costs, testing of subsequent pathogens would not proceed if a previous test was positive. This eliminated any detection of combined viral infection. There was no plan to investigate whether the disease could be an opportunistic infection of HIV.

For the bacterial pathogens, we consulted microbiology websites and Pubmed indexed articles to check whether the reported pathogens had ever caused meningitis or bacteremia. Pathogens which had never been reported as etiology of AME were excluded.

### Quality control

Before and during the study period, training was given to personnel involved at all levels. The hospitals were visited every 10 days and all potential cases reviewed to validate the reports. All the laboratory techniques were supervised by World Health Organization, US CDC and China CDC. Guangxi CDC and China CDC randomly selected and tested 10% of samples that were tested in Guigang CDC.

### Data analysis

R-software 2.14.0 was used for data analysis. Yearly incidences of AME, JE, measles, mumps, rubella, other viruses, bacteria and fungi were computed. The Kruskal-Wallis and Wilcoxon's rank sum tests were used to compare the hematological/biochemistry test results. Six hematological and biochemical variables were highly correlated. Including all of them in the multivariate model will mutually cancel out the effects of individual variables. Factor analysis was used to obtain a fewer number of uncorrelated factor scores to be used as independent variables. A multinomial logistic regression model was then fit to the outcome categorized into four groups: negative (reference group), JE, other viruses and bacteria/fungi. The potential independent variables included the hematological/biochemical factor scores, age and clinical manifestations. Akaike's information criterion (AIC) was used as the indicator to choose the best model at each step using a backwards elimination strategy. Finally, due to small sample size of many etiologic agents, we compared bacterial/fungal infections, which are treatable, to all viral agents + the negative group, which have no specific treatment. This binary outcome was modeled against predictors among demographic and clinical variables. The area under the receiver operating characteristic (ROC) curve was used to quantify the discriminatory ability of this model. Statistical significance was set at 0.05 for all analyses.

### Ethical considerations

This study was approved by the Ethical Review Committee of China CDC [8]. Written informed consent was obtained from the study subjects as routine in hospital before CSF specimens were collected. If the study subjects were aged less than 18 years, written informed consent was obtained from their parents or guardians.

## Results

### General information

The number of suspected cases reported from May 2007 to June 2012 was 2,494. After excluding 112 cases confirmed to be caused by other diseases, 2,382 probable cases with 75 fatalities were included (case-fatality rate 3.1%). The numbers of cases with blood and CSF specimens were 2,375 and 1,887, respectively.

**Fig 1:**
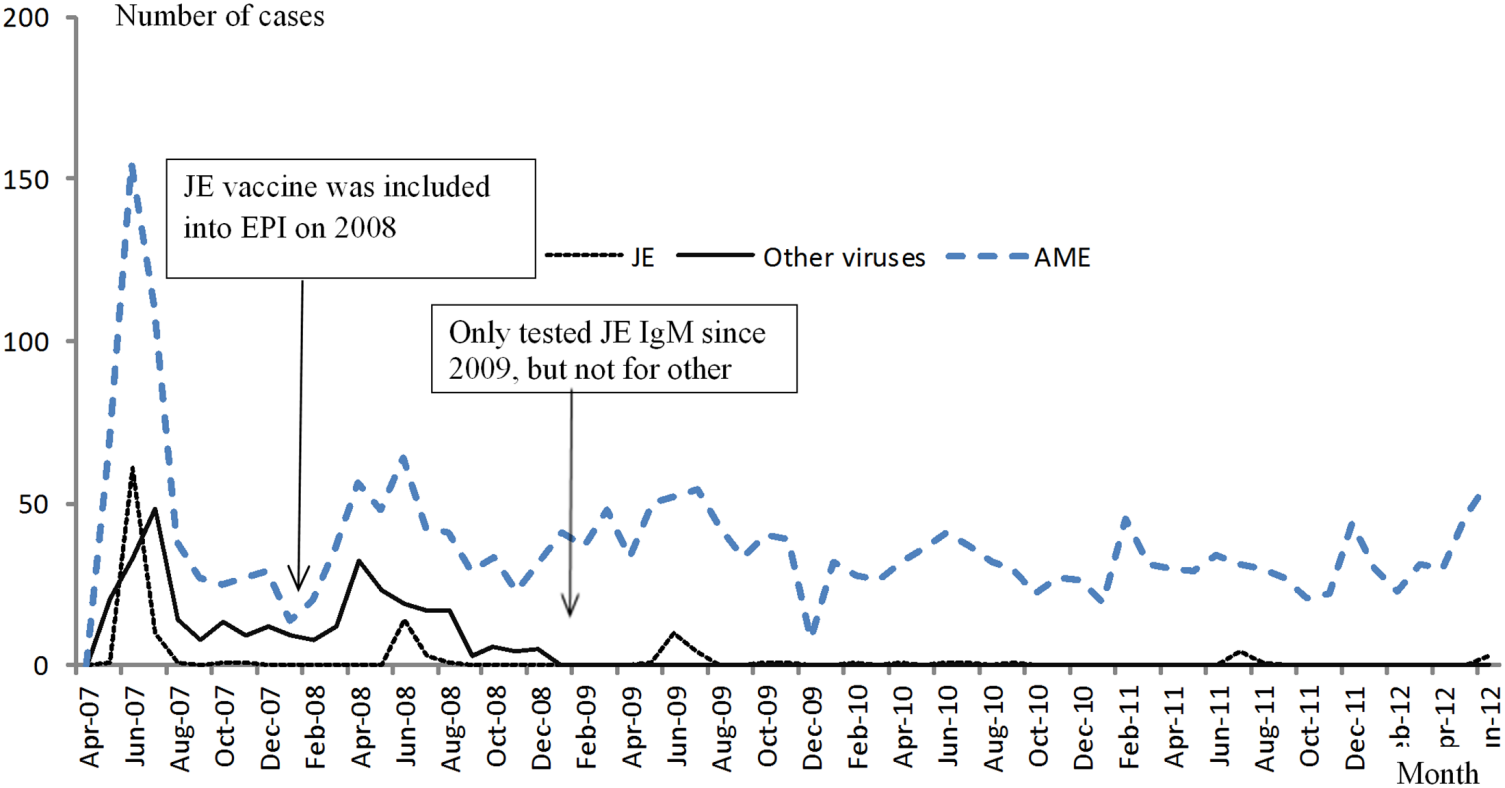
Monthly distribution of acute meningitis and encephalitis (AME) cases, Japanese encephalitis (JE) and other viruses in Guigang City, Guangxi, May 2007—June 2012. *Only those cases with negative JE IgM results and onset between May 2007 and December 2008 were tested for other viruses.

A total of 2,168 cases were tested for JE. Of these, 123 were positive. Among the 2,045 JE negative cases, 724 cases whose onset was between May 2007 and December 2008 were tested for other viral agents and 312 were positive. Of 1,927 cases with results on bacterial culture and/or PCR tests available, 103 were positive for at least one infection. Table 1 and Fig 1 summarize the yearly number and incidence rate of AME and cases with known etiology. The yearly incidence rate of AME was relatively stable between 2007 and 2012 while JE sharply dropped after 2007 when JE vaccine became a part of EPI. JE and other viruses exhibited a seasonal pattern with a summer peak from April to August, during which the number of cases accounted for 94.3% and 71.5% of annual cases, respectively. Information for other viruses was available only in the first two years. One of the five counties was removed from the study after 2010 due to budget limitations. The overall incidence of bacterial AME was higher than that of JE after 2007.

**Table 1:** Yearly incidence rate of acute meningitis and encephalitis (AME) in Guigang City, Guangxi, May 2007- June 2012.

Year	Population	No. of AME probable cases (/100,000 population)ᶤ	No. of positive (/100,000 population)^ᶤ^		
			Japanese encephalitis	Other viruses^ᶠ^	Bacteria/fungi
2007	4113366	481 (14.95)	75 (1.85)	157 (5.42)	23 (0.75)
2008	4159495	438 (10.53)	18 (0.43)	155 (3.73)	18 (0.51)
2009	4207080	511 (12.15)	17 (0.40)	-	27 (0.69)
2010*	3084655	371 (12.02)	4 (0.13)	-	19 (0.68)
2011	2990779	365 (12.20)	5 (0.17)	-	13 (0.53)
2012	3012618	216 (13.51)	4 (0.20)	-	3 (0.28)
Annual incidence rate		2382 (12.55)	123 (0.58)	312 (4.57)	103 (0.59)

^ᶤ^ The number in parentheses is the incidence rate (per 100,000 population). As the surveillance started in May 2007 and ended in June 2012, the incidence was seasonally adjusted using data from 2008 to 2011. The pathogen specific incidence rate was adjusted by the proportion of incomplete laboratory testing due to not all AME cases being tested for pathogens.

* One county was excluded from the study area since 2010 due to the budget limitations.

^ᶠ^ Other viruses IgM were only tested in 2007 and 2008.

The frequency of detected pathogens is shown in Table 2. The top three causative viruses were enteroviruses, mumps virus and JE virus, together accounting for more than 75% of viral pathogens identified. AME caused by enterovirus had the highest annual incidence rate (2.01 per 100,000 population). Measles, mumps and rubella had annual incidence rates of 0.32, 1.48 and 0.16 per 100,000 population, respectively. The annual incidence rate of AME caused by HSV, CMV, VZV and EB viruses per 100,000 population were 0.29, 0.15, 0.12 and 0.04, respectively. There were 78 bacterial meningitis cases. Of these, 22 cases were caused by three vaccine-preventable pathogens (*Sp*, *Nm and Hib*), and 56 cases by 31 other bacteria. The leading etiologic bacteria were *Streptococcus sp*. (27) and *Staphylococcus sp*. (18). Of 25 patients infected with fungi, 22 were infected with *Cryptococcus neoformans*, nineteen of whom were adults. The annual incidence rate of *Sp*, *Nm*, *Hib*, other *Streptococcus sp*., *Staphylococcus sp*. and *Cryptococcus neoformans* per 100,000 population were 0.09, 0.02, 0.02, 0.15, 0.06 and 0.13, respectively.

**Table 2:** Frequency of acute meningitis and encephalitis pathogens in Guigang City, Guangxi, May 2007- June 2012*.

Viruses (n = 435)ᶠ	Bacteria (n = 78)		Fungi (n = 25)
Japanese encephalitis (123)	Streptococcus pneumonia (16)	Staphylococcus hominis (4)	Cryptococcus neoformans (22)
Enterovirus (137)	*Neisseria meningitis* (3)	Micrococcus luteus (2)	Candida tropicalis (1)
Mumps (101)	Haemophilus influenza type B (3)	Sphingomonas paucimobilis (2)	Canidia albicans (1)
Measles (22)	Streptococcus suis (7)	Staphylococcus xylosus (2)	Pichia ohmeri (1)
Herpes simplex virus (20)	Staphylococcus epidermidis (5)	Agrobacterium radiobacter (1)	
Rubella (11)	Escherich spp or E. coli (5)	Chryseobacterium indologenes (1)	
Cytomegalovirus (10)	Klebsiella pneumonia (4)	Corynebacterium spp (1)	
Varicella-zoster virus (8)	Staphylococcus aureus (2)	Hafinia alvei (1)	
EB virus (3)	Staphylococcus haemolyticus (2)	Kocuria roseus (1)	
	Group A Strep (1)	Kocuria varians (1)	
	Pseudomonas aeruginosa (1)	Moraxella lacunata (1)	
	Pseudomonas stutzeri (1)	Ochrobactrum anthropi (1)	
	Salmonella Paratyphi b (1)	Sphingobacterium multivorum (1)	
	Salmonella Typhi (1)	Staphylococcus capitis (1)	
	Stenotrophomonas maltophilia (1)	Staphylococcus chomogenes (1)	
	Streptococcus intermedius (1)	Staphylococcus warneri (1)	
	β hemolytic streptococci (1)	Streptococcus sanguis (1)	

* Number in bracket is the positive number.

^ᶠ^ Japanese encephalitis IgM were tested in the whole study period while other viruses IgM were only tested in 2007 and 2008.

### Case characteristics and clinical features

All three *Hib* cases were male and aged less than 5 years. Among the three *Nm* cases, 2 were male and 1 was female; 2 were aged between 5 and 17 years and 1 was aged more than 18 years. All cases were either cured or improved on discharge from hospital. Tables 3 and 4 summarize the characteristics and clinical features of the AME patients. There were more males than females. Viruses mainly affected young children and adolescents while bacteria and fungi were more common in adults. Fungal meningitis had the highest case fatality rate (8%), followed by bacterial meningitis (6.2%—6.5%). Infection by JE and bacteria/fungi had more severe clinical manifestations, such as lethargy, drowsiness and alteration of consciousness. These infections also increased white blood cell, blood neutrophils and CSF leukocyte counts. However, only bacteria/fungi infection increased CSF protein.

**Table 3:** General characteristics of acute meningitis and encephalitis (AME) cases, Japanese encephalitis (JE), other viral encephalitis, fungi, and bacterial meningitis in Guigang City, Guangxi, May 2007- June 2012.

	AME cases (n = 2382)	Negative (n = 246)ᶤ	Laboratory test results				
			Viruses		fungi (n = 25)	Bacteria	
			JE (n = 123)	Other viruses (n = 312)		S. pneumoniae (n = 16)	Other bacteria (n = 62)
**Gender**							
Male	1519 (63.8)	174 (70.7)	68 (55.3)	173 (55.4)	17 (68.0)	10 (62.5)	43 (69.4)
Female	863 (36.2)	72 (29.3)	55 (44.7)	139 (44.6)	8 (32.0)	6 (37.5)	19 (30.6)
**Age** *	14.3±20.7	23.3±25.8	4.4±5.1	12.2±17.5	42.2±22.5	16.8±25.9	22.0±26.4
<5y	1216 (50.9)	90 (36.6)	95 (77.2)	168 (53.8)	0	10 (62.5)	31 (50.0)
5-17y	627 (26.3)	57 (23.2)	24 (19.5)	85 (27.2)	5 (20.0)	2 (12.5)	9 (14.5)
≥18y	539 (22.6)	99 (40.2)	4 (3.3)	59 (18.9)	20 (80.0)	4 (25.0)	22 (35.5)
**Prognosis**							
Cure/better	2210 (92.8)	222 (90.2)	109 (88.6)	287 (92.0)	20 (80.0)	14 (87.5)	51 (82.3)
Not improve	97 (4.1)	16 (6.5)	10 (8.1)	9 (2.9)	3 (12.0)	1 (6.2)	7 (11.3)
Death	75 (3.1)	8 (3.3)	4 (3.3)	16 (5.1)	2 (8.0)	1 (6.2)	4 (6.5)

* Values are mean ± standard deviation.

^ᶤ^ Only include those test both virus and bacteria.

**Table 4:** Comparison of clinical features of negative group, Japanese encephalitis, other viral encephalitis and bacterial meningitis in Guigang City, Guangxi, May 2007- June 2012.

Clinical features	Negative (n = 246)	JE (n = 123)	Other viruses (n = 312)	Bacteria/fungi (n = 103)	P-value
Fever	222 (90.2)	120 (97.6)	288 (92.3)	97 (94.2)	0.1
Degree of fever^ᶤ^	137 (61.7)	82 (68.3)	109 (37.8)	54 (55.7)	**<0.01**
Headache	108 (43.9)	30 (24.4)	108 (34.6)	54 (52.4)	**<0.01**
Diarrhea	13 (5.3)	9 (7.3)	30 (9.6)	11 (10.7)	0.2
Vomiting	113 (45.9)	57 (46.3)	134 (42.9)	62 (60.2)	**0.02**
Projectile vomiting	9 (8.0)	2 (3.5)	9 (6.7)	5 (8.1)	0.46
Lethargy	114 (46.3)	86 (69.9)	146 (46.8)	67 (65.0)	**<0.01**
Drowsiness	70 (28.5)	73 (59.3)	87 (27.9)	47 (45.6)	**0.02**
Alteration of Consciousness	66 (26.8)	61 (49.6)	79 (25.3)	50 (48.5)	**<0.01**
Convulsion	113 (45.9)	100 (81.3)	160 (51.3)	47 (45.6)	**<0.01**
Neck stiffness	49 (19.9)	35 (28.5)	39 (12.5)	30 (29.1)	**<0.01**
Opisthotonus	5 (2.0)	13 (10.6)	7 (2.2)	5 (4.9)	**<0.01**
Meningeal irritation	57 (23.2)	40 (32.5)	48 (15.4)	37 (35.9)	**<0.01**
Skin petechiae	1 (0.4)	3 (2.4)	5 (1.6)	3 (2.9)	0.13
White blood cell (×10^9^)*	9.8 (7.0–14.1)	17.5 (12.5–22.0)	9.4 (7.3–14.4)	11.9 (7.2–17.5)	**<0.01**
Blood neutrophils (%)*	70.0 (55.7–80.0)	77.7 (64.7–84.0)	67.8 (51.2–78.9)	76.3 (62.5–85.1)	**0.01**
CSF protein, g/d*	0.35 (0.15–0.9)	0.36 (0.18–0.52)	0.30 (0.17–0.50)	0.48 (0.23–1.34)	**<0.01**
CSF leukocyte count (×10^6^)*	8.5 (4.0–29.3)	25.0 (8.0–110.0)	9.0 (5.0–30.0)	63 (10–305)	**<0.01**
CSF glucose (mmol/L)*	3.5 (2.9–4.3)	4.2 (3.3–5.0)	3.7 (3.0–4.4)	2.5 (0.9–3.9)	**<0.01**
CSF chloride (mmol/L)*	116 (109–120)	118 (110–121)	116 (110–120)	118 (110–123)	0.27

^ᶤ^ Body temperature > 39°C

*Median (interquartile range) with P-value of Kruskal-Wallis rank sum test

### Factor analysis and multinomial logistic regression

541 cases with viral and bacterial culture/PCR results were included in the factor analysis of the hematological/biochemical measurements. Two factors were identified (Table 5). We named factor 1 as leukocytosis and factor 2 as CSF inflammation.

**Table 5:** Exploratory factor analysis result by using blood and cerebrospinal fluid (CSF) biochemistry test results from 541 cases.

	Factor1*(leukocytosis)	Factor2*(CSF inflammation)
Blood white cell (×10^9^)	0.994	
Blood neutrophils (%)	0.265	
CSF protein	0.115	0.581
CSF leukocyte count (×10^6^)	0.128	0.582
CSF glucose (mmol/L)		-0.432
CSF chloride (mmol/L)		

* The factor loadings value; the rotation method was varimax; the cumulative variance of factor1 and factor 2 was 32.8%.

A multinomial logistic regression with age, clinical manifestations and factor scores of leukocytosis and CSF inflammation as independent variables was conducted (Table 6). Age, alteration of consciousness, leukocytosis and CSF inflammation were the only independent and statistically significant factors predicting AME. Compared to the negative group, JE and other viruses occurred at a younger age. Both JE (OR) and bacteria/fungi infections were more likely to cause alteration of consciousness and leukocytosis compared to negative cases. Only bacteria/fungi infection increased CSF inflammation. When combining the negative group with JE and other viral agents (as reference group), leukocytosis became non-significant. The area under the ROC curve was 72.12% indicating good but imperfect prediction (Table 7).

**Table 6:** Final multinomial logistic regression model predicting AME. Negative cases (n = 176) are the reference group.

	Japanese encephalitis (n = 99) OR (95% CI)	Other viruses (n = 186) OR (95% CI)	Bacteria/fungi (n = 80) OR (95% CI)	P-value*
Age (years)				<0.001
<5	1	1	1	
5–17	0.67 (0.34, 1.32)	1.12 (0.64, 1.97)	1.20 (0.54, 2.66)	
≥18	**0.05 (0.02, 0.16)**	**0.41 (0.25, 0.69)**	0.90 (0.46, 1.74)	
Alteration of consciousness	**2.14 (1.18, 3.91)**	0.98 (0.59, 1.62)	**2.31 (1.26, 4.22)**	0.001
Leukocytosis	**2.28 (1.66, 3.13)**	1.07 (0.80, 1.42)	**1.24 (1.02, 1.95)**	<0.001
CSF inflammation	0.93 (0.71, 1.22)	0.85 (0.68, 1.05)	**1.49 (1.22, 1.81)**	<0.001

*Likelihood ratio test. OR: odds ratio. CI: confidence interval.

**Table 7:** Final logistic regression model by using factor scores*.

	Crude OR (95% CI)	Adjusted OR (95% CI)	P-value
Age			<0.001
<5y	1	1	
5-17y	1.15 (0.59, 2.24)	1.31 (0.65, 2.64)	0.454
≥18y	2.36 (1.38, 4.05)	**1.95 (1.08, 3.53)**	0.028
Alteration of consciousness	1.37 (0.84, 2.67)	**1.96 (1.15, 3.36)**	0.014
CSF inflammation	1.65 (1.42, 1.93)	**1.63 (1.39, 1.91)**	<0.001

*Negative cases, JE and other viral agents combined are the reference group

## Discussion

This study revealed that AME cases were more common in summer. The majority of etiologic agents for AME in the study areas were viruses. Immunization has been effective in controlling JE. Emerging enteroviruses and certain vaccine preventable viruses are still a major problem. To a lesser extent, certain AME cases were also caused by vaccine preventable bacteria and *Cryptococcus neoforman*. Others were miscellaneous agents. Compared to negative cases, both JE and other viruses infection occurred in lower age groups. Leukocytosis and alteration of consciousness were more common in JE and bacterial/fungal infections. These clinical manifestations and routine laboratory findings, however, was far from perfect in predicting the etiologic agents.

More than 100 organisms can cause viral encephalitis [5]. HSV was the most common causative virus reported in the United States [13]. A hospital based study in Taiwan showed that HSV was the predominant pathogen (36%), followed by VZV (13%) [14]. In our study, enteroviruses, mumps and JE viruses were the top three causative viruses with a summer peak from April to August. Enterovirus predominance in the study area is consistent with the high incidence of hand, foot, and mouth disease (HFMD) in Guangxi and other parts of China. The incidence rate of HFMD has sharply increased since it was categorized as a notifiable disease in May 2008 and substantial cases with neurological complications have been reported [15,16]. Similar to our finding, studies in England reported that enterovirus was the most common virus in the post MMR vaccine era [17–19]. On the other hand, we showed evidence that JE incidence was reduced after JE vaccine was included into EPI in 2008. The effects of introducing mumps and rubella vaccines will need to be further assessed.

Although the measles vaccine has been covered in the EPI since 1978 and the reported coverage rate has been over 90% in Guangxi [20], measles encephalitis is not rare in the study area. Moreover, many outbreaks have been reported in Guangxi and China since 2013 [21]. The real coverage rate and the effectiveness of the measles vaccine should therefore be further investigated.

Acute bacterial meningitis is the most severe type of meningitis. The incidence rate has decreased and the predominant pathogens have changed in many countries with the introduction of *Nm* vaccines, *Hib* vaccine and heptavalent pneumococcal conjugated vaccine (PCV 7) [22–28]. In the United States, the rate of bacterial meningitis among children < 5 years declined by 55% in the early 1990s when *Hib* vaccine for infants was introduced [23]. The median (IQR) incidence per 100,000 child years in Western Pacific region was 42.9 (12.4–83.4) and in South East Asia was 26.8 (21.0–60.3) [29]. In China, *Hib* and PCV 7 vaccines are not included in the EPI. As China has a steady economic growth, the cost-effectiveness of these vaccines should be reviewed from time to time.

*Cryptococcus neoformans* is the most common fungal infection of the CNS [30]. This pathogen was rarely found in China in the past. With the emergence of HIV/AIDS and the expanded use of immunosuppressive drugs, *Cryptococcus neoformans* incidence has been increasing steadily with an estimated 140,000 new infections annually in Asia [31]. In our study, *Cryptococcus neoformans* was identified in 22 patients, 19 of whom were adults. Hebei province, also involved in the AMES project, found 23 cases of cryptococcal meningitis during a similar study period, all of which were children aged <18 years and all were HIV-negative [32]. Whether the cryptococcal infections detected in our study were on top of HIV infection is unknown.

As fewer than half of AME cases revealed causative agents [11,33,34], clinicians often need to diagnose patients according to symptoms, signs or hematological /biochemistry results. Classically, it is assumed that the white blood cell, neutrophils, CSF protein and CSF leukocyte count in bacterial meningitis are usually higher than in viral encephalitis, but CSF glucose is lower [35,36]. We confirmed that bacterial/fungal infections were more likely to cause leukocytosis and CSF inflammation compared to negative cases. However, JE is also more likely to cause leukocytosis, but not CSF inflammation. Finally, leukocytosis was ruled out in the model of distinguishing aseptic from bacterial infection. However, as the area under the ROC curve was only 72.1%, bacterial culture cannot be omitted from diagnostic testing for suspected AME cases.

## Limitations

Despite a large sample size and the study being conducted in a previous JE endemic area, which increases the generalizability of our results, there are some limitations which should be mentioned. The ELISA test for IgM antibodies for many viral infections is not the gold standard. The tests were carried out sequentially, stopping whenever a positive result was found, a process which cannot detect combined infections. These viral investigations were confined to 2007 and 2008 making trend analysis of viral AME rather limited. The positive rate of bacteria culture was low, which may be due to the prior antibiotic use or inadequate culture technique [13].

## Conclusion

A wide range of organisms were identified in the study areas. Clinical features and routine laboratory tests imperfectly discriminate bacterial from aseptic AME. Bacterial culture is therefore obligatory in AME patient care. JE had been controlled by vaccines, yet a significant proportion of cases were infected by vaccine preventable organisms. This suggests the need to further review the JE vaccine policy.

## Supporting Information

S1 Dataset (XLSX)
